# Spin-orbit torque–driven propagating spin waves

**DOI:** 10.1126/sciadv.aax8467

**Published:** 2019-09-27

**Authors:** H. Fulara, M. Zahedinejad, R. Khymyn, A. A. Awad, S. Muralidhar, M. Dvornik, J. Åkerman

**Affiliations:** 1 Physics Department, University of Gothenburg, 412 96 Gothenburg, Sweden.; 2 NanOsc AB, Electrum 229, 164 40 Kista, Sweden.; 3 Material and Nanophysics, School of Engineering Sciences, KTH Royal Institute of Technology, Electrum 229, 164 40 Kista, Sweden.

## Abstract

Spin-orbit torque (SOT) can drive sustained spin wave (SW) auto-oscillations in a class of emerging microwave devices known as spin Hall nano-oscillators (SHNOs), which have highly nonlinear properties governing robust mutual synchronization at frequencies directly amenable to high-speed neuromorphic computing. However, all demonstrations have relied on localized SW modes interacting through dipolar coupling and/or direct exchange. As nanomagnonics requires propagating SWs for data transfer and additional computational functionality can be achieved using SW interference, SOT-driven propagating SWs would be highly advantageous. Here, we demonstrate how perpendicular magnetic anisotropy can raise the frequency of SOT-driven auto-oscillations in magnetic nanoconstrictions well above the SW gap, resulting in the efficient generation of field and current tunable propagating SWs. Our demonstration greatly extends the functionality and design freedom of SHNOs, enabling long-range SOT-driven SW propagation for nanomagnonics, SW logic, and neuromorphic computing, directly compatible with CMOS technology.

## Teaser

Propagating spin waves generated by a metal-based spin Hall nano-oscillator for highly energy-efficient spin wave technology.

## INTRODUCTION

The recent emergence of spin-orbit torque (SOT) (*1*) from pure spin currents has opened an additional avenue for the controlled manipulation of magnetic moments in spintronic devices, resulting in markedly improved efficiency (*2*) and much lower power dissipation (*3*) compared to conventional spin-transfer torque (*4*). Thanks to the ability of SOT to compensate natural magnetic damping over spatially extended regions, there has been considerable interest in exploring the long-range enhancement of spin wave (SW) propagation (*5*–*8*) in a variety of nanoscale devices with the aim of developing an energy-efficient and ultrahigh-speed beyond–complementary metal-oxide semiconductor (CMOS) SW-based technology for signal processing (*9*) and computation (*10*, *11*)—so-called magnonics (*12*, *13*).

One of the most promising SOT devices for active, controllable SW generation on the nanoscale is the nanoconstriction-based spin Hall nano-oscillator (SHNO) (*14*–*18*). It can be easily fabricated using a wide range of different bilayer combinations (*14*, *16*, *19*), and the generated SWs can be directly observed using both electrical and optical microwave spectroscopy (*16*, *19*, *20*). For applications, nanoconstriction SHNOs exhibit highly robust mutual synchronization, both in long chains (*16*) and in two-dimensional arrays (*21*), which both improves their signal properties by orders of magnitude and lend themselves to neuromorphic computing (*22*, *23*).

A major limitation of nanoconstriction SHNOs, however, is the localized nature of the SOT-driven auto-oscillations (*17*, *24*). The localization is a consequence of the easy-plane anisotropy and the geometry of the device, which lead to a negative magnetodynamic nonlinearity, further exacerbated by the Oersted field from the drive current and from the SOT itself (*24*). It would be highly advantageous if the localization could be mitigated so as to generate truly propagating SWs. Not only should this lead to mutual synchronization over much longer distances, but it would also make SOT-driven SWs directly applicable to additional nonconventional computing schemes such as wave-based computing (*10*, *13*, *25*).

In a recent work, Evelt and co-workers (*20*) demonstrated SOT-driven propagating SWs in extended Bi-substituted yttrium iron garnet films with perpendicular magnetic anisotropy (PMA). While the auto-oscillations could only be observed optically and did not exhibit any frequency tunability via the drive current, the demonstration raises the question whether the addition of PMA to metal-based nanoconstriction SHNOs could potentially lead to SOT-generated propagating SWs in more practical devices directly compatible with CMOS technology (*19*). Here, we show, using 150- and 200-nm nanoconstrictions in W/CoFeB/MgO material stacks with substantial PMA, that it is possible to generate strongly current-tunable propagating SWs over a very wide frequency range of about 3 to 22 GHz. The SWs are studied using electrical microwave spectroscopy and modeled using micromagnetic simulations. Auto-oscillations are observed at currents as low as 0.15 mA, where they are still localized and exhibit negative nonlinearity. As the current is increased, the nonlinearity changes sign and the localized SWs exhibit smooth transition into propagating SWs at about 0.5 mA. It is hence possible to seamlessly turn on and off the localization, which will allow the generation of ultrashort SW pulses driven by the current alone, which is much faster than using external fields (*26*).

### PMA controlling the magnetodynamic nonlinearity

The rich nonlinear magnetodynamics in patterned magnetic thin films can be analytically described by a single nonlinearity coefficient, N, the magnitude and sign of which determine the strength and nature of magnon-magnon interactions, with positive and negative values signifying magnon repulsion and attraction, respectively (*24*, *27*). As spin-transfer torque and SOT can generate very high SW amplitudes, the sign and magnitude of N leads to distinctly different behavior of the auto-oscillations. A negative nonlinearity makes the auto-oscillation frequency decrease with amplitude, eventually moving it into the magnonic bandgap, where it first leads to SW self-localization, and can further promote the nucleation of magnetodynamical solitons such as SW bullets (*28*, *29*) in easy-plane magnetic films and magnetic droplets (*30*, *31*) in films with very large PMA. A large positive nonlinearity, on the other hand, makes the auto-oscillation frequency increase with amplitude to well above the ferromagnetic resonance (FMR) frequency, leading to the propagation of SWs with a finite real wave vector. A prominent example is the so-called spin-torque–driven Slonczewski modes (*32*–*34*), which can also form SW beams in oblique fields, particularly useful for mutual synchronization (*34*).

**Fig. 1:**
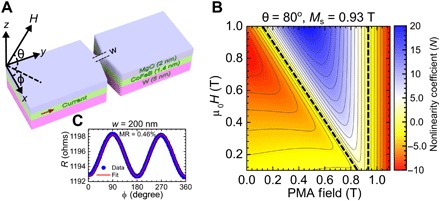
Device schematic, magnetoresistance, and nonlinearity coefficient. (**A**) Schematic of a SHNO with nanoconstriction width *w*. (**B**) Contour plot displaying the analytically calculated nonlinearity coefficient (N) as a function of PMA strength and applied OOP (θ = 80°) field for a thin magnetic film with saturation magnetization, μ_0_*M*_S_ = 0.93 T; dashed black line indicates N=0. (**C**) Anisotropic magnetoresistance (MR) measured with 0.05 mA on a 200-nm nanoconstriction under a rotating 70 mT in-plane (IP) field.

The nonlinearity is generally governed by the applied field vector and/or an effective magnetic anisotropy tensor and is zero for an isotropic magnet regardless of the applied external field strength. The easy-plane shape anisotropy of a magnetic thin film holds the magnetization vector in the film plane; therefore, the nonlinearity strongly depends on the strength and orientation of the applied field. However, anisotropy induced by an interface to a different material can counteract the shape anisotropy and even pull the magnetization vector out of the film plane. This PMA contributes a term with the opposite sign in the nonlinear coefficient compared to the shape anisotropy. The impact of PMA on the sign and strength of N can then be calculated for a thin magnetic film using the method given in (*27*), with the result plotted in Fig. 1B as a function of the strength of applied out-of-plane (OOP) field (θ_ex_ = 80°) and the PMA field.

One notes that N increases monotonically from negative (red regions) to positive (blue regions) values as a function of the OOP field strength and goes through zero at a certain field value (black dashed line) that depends on the PMA strength (*29*); that is, the PMA shifts the point of zero nonlinearity toward lower values of applied field. Adding PMA hence makes it possible to reach positive nonlinearity at much lower fields. As the auto-oscillation threshold current decreases with decreasing field, it should, in principle, be possible to drive propagating SWs at much lower currents, which the SHNO can sustain without degradation.

As a side note, any further increase of the PMA beyond the point where it completely compensates the shape anisotropy, i.e., where the magnetization equilibrium angle changes from in-plane (IP) to perpendicular to the plane, again results in a negative N (the second red region at high PMA in Fig. 1B). As a consequence, the current dependence of the auto-oscillation frequency again becomes negative, and the generated SWs self-localize and can eventually nucleate magnetic droplet solitons (*30*, *31*, *35*).

## RESULTS

### Nanopatterned SHNO device schematic and magnetodynamics

A schematic of a nanoconstriction SHNO is shown in Fig. 1A. The material stack consisted of sputtered β–W(5 nm)/Co_20_Fe_60_B_20_(1.4 nm)/MgO(2 nm). The β-phase of W has been shown to produce large SOT (*36*), and a thinner CoFeB interfaced with MgO layer enhances PMA in the CoFeB layer (*37*). The stack was fabricated on highly resistive Si substrates to both dissipate the local heat generated during operation and to reduce microwave losses; the SHNOs are hence CMOS compatible (*19*). A positive dc is injected from the signal pad to ground along the *y* direction, while φ and θ define the IP and OOP field angles, respectively. Figure 1C shows the IP angular dependence of the magnetoresistance (MR) measured for a 200-nm-wide nanoconstriction SHNO, exhibiting a relatively large overall MR value of 0.46% between the parallel and perpendicular orientation; the angular-dependent MR is known to have contributions both from anisotropic MR and spin Hall MR (*38*).

**Fig. 2:**
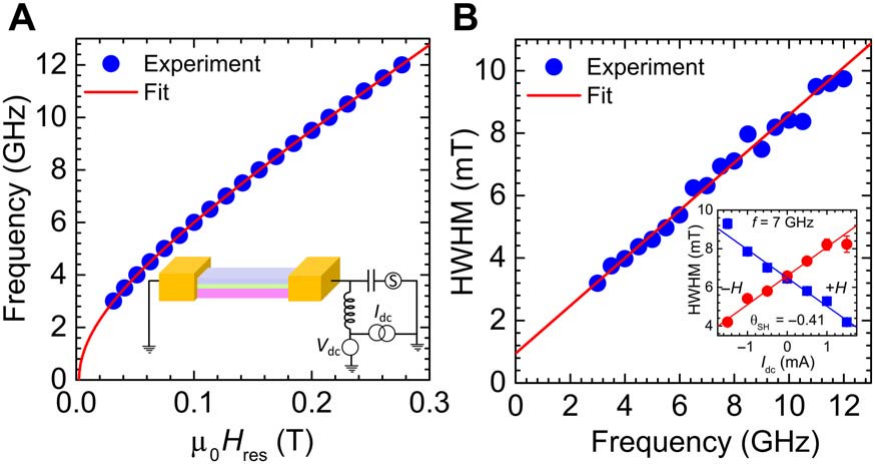
ST-FMR measurements. (**A**) Resonance frequency versus IP field (blue dots) with a Kittel fit (red line) yielding an effective magnetization $\mu_{0}M_{\text{eff}}=\mu_{0}(M_{s}-H_{k}^{⊥})$ of 0.31 T. Inset: Illustration of the ST-FMR measurement on a microstrip with dimensions of 6 μm by 18 μm. (**B**) Extracted linewidth (HWHM) versus resonance frequency yielding a Gilbert damping constant of α = 0.023. Inset: Current-dependent ST-FMR linewidth for both positive (blue squares) and negative (red dots) field directions yielding an SHA of −0.41.

Figure 2 summarizes the spin-torque FMR (ST-FMR) measurements performed on a microstrip of W/CoFeB/MgO with dimensions of 6 μm by 18 μm to determine the magnetodynamical parameters. The inset of Fig. 2A schematically illustrates the experimental setup used for the ST-FMR (see Materials and Methods). The main panel of Fig. 2A shows the extracted resonance peak positions obtained at different microwave frequencies ranging from 3 to 12 GHz under radio frequency (RF) current excitation. The resonance field dependence on frequency can be well fitted with the Kittel equation, yielding an effective magnetization μ_0_*M*_eff_ = 0.31 T with a gyromagnetic ratio of γ/2π = 29.9 GHz/T. With the saturation magnetization, μ_0_*M*_S_ = 0.93 T obtained from alternating gradient magnetometry measurements, we extract a PMA field of $\mu_{0}H_{k}^{⊥}=\mu_{0}(M_{S}-M_{\text{eff}})=$ 0.62 T; that is, while we have substantial PMA, it is not strong enough to pull the magnetization OOP. Figure 2B displays the plot of linewidths extracted as half width at half maximum (HWHM) from the same resonance peaks as a function of different microwave frequencies, and the linear best fit of experimental data gives rise to a Gilbert damping constant of α = 0.023. The inset of Fig. 2B shows the current-induced linewidth changes extracted at a fixed microwave frequency of 7 GHz for two opposite IP field orientations, the slope of which yields a high value of the spin Hall angle (SHA), θ_SH_ = −0.41, typical for β-W and indicating the presence of large SOT from the spin Hall effect in our devices (*36*).

### Propagating SWs

**Fig. 3:**
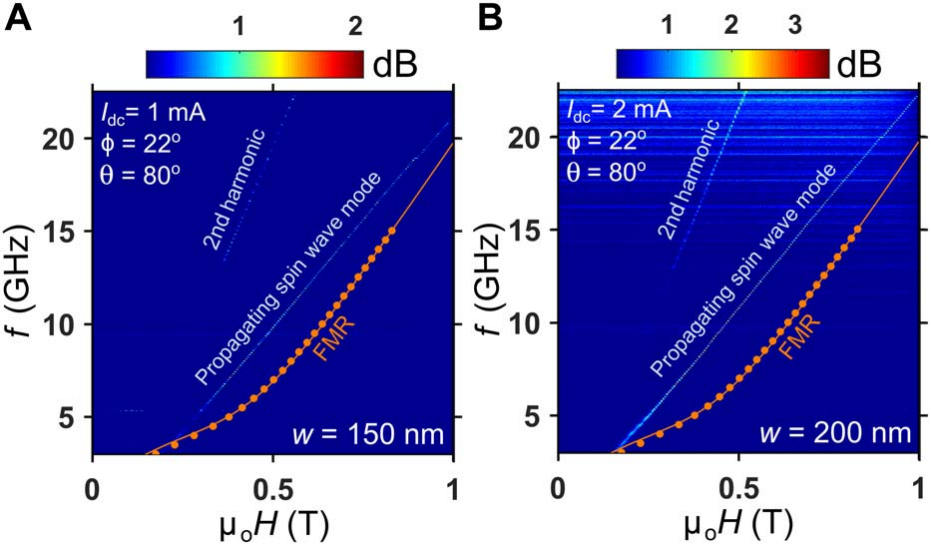
Auto-oscillating propagating SWs versus OOP field strength. PSDs versus OOP field for a (**A**) 150-nm and (**B**) 200-nm nanoconstriction. Orange data points are the ST-FMR resonances obtained under identical conditions on a microstrip with dimensions of 4 μm by 14 μm with the solid line being a fit to the Kittel equation (Eq. 2 in Materials and Methods).

Figure 3 shows color plots of the generated microwave power spectral density (PSD) as a function of OOP applied field strength measured for two different nanoconstriction widths with fixed direct currents of *I*_dc_ = 1 and 2 mA, respectively. In both measurements, the IP field angle was fixed at φ = 22° to ensure sufficient electrical sensitivity to the auto-oscillation signal, while the OOP field angle θ = 80° was chosen in a way to achieve large positive nonlinearity in the active nanoconstriction region. The orange circles, fitted with a solid orange line using the Kittel equation (Eq. 2), represent the FMR frequencies obtained from ST-FMR measurements on microstrips under identical applied field conditions. As the FMR frequency corresponds to a wave vector of $\vec{k}$ = 0, it allows us to distinguish between propagating and localized SW modes in the SHNO since spatially localized modes with a frequency well below FMR have no well-defined real wave vector, while propagating modes with frequency higher than the FMR have a finite real $\vec{k}$.

Note that in all previously investigated nanoconstriction and nanogap SHNOs (*16*, *17*, *39*), the auto-oscillations remained localized with a frequency lower than the FMR spectrum of the magnetic material. As can be seen in Fig. 3A, this is, in our case, only true for μ_0_*H* < 0.2 T, and at all higher fields, the auto-oscillation frequency lies up to several gigahertz above the FMR frequency. This general behavior was observed in different devices, as shown in Fig. 3B, where a larger nanoconstriction with *w* = 200 nm follows the same trend but now with improved microwave characteristics. Note that, in addition to a higher output power, the auto-oscillations in the larger nanoconstriction cross over into propagating SWs already at μ_0_*H* ∼ 0.15 T, indicating a higher PMA than in the smaller nanoconstriction. This is a general trend for different nanoconstriction widths, which we believe is an effect of an etch-induced reduction of PMA at the nanoconstriction edges, which affects the smaller nanoconstrictions in greater proportion.

The field dependence in Fig. 3 is entirely consistent with the expected behavior based on Fig. 1B, where the influence of the PMA field strength on the nonlinearity (N) is depicted. In weak magnetic fields, N is negative, leading to localization of the auto-oscillations, but in stronger fields, N changes sign to positive, resulting in propagating SWs. The strong PMA allows one to achieve a positive N at a lower OOP magnetization angle θ_M_, which, in turn, effectively results into a higher spin Hall efficiency via sin(90° − θ_M_) dependence and, therefore, substantially reduces the operational current (*40*). With a strong PMA field due to thinner CoFeB layer in the present case, the lowest threshold current density of auto-oscillations is obtained as 1.5 × 10^7^ A/cm^2^, which is considerably lower compared to the one observed in our recent study on relatively thicker CoFeB-based W/CoFeB/MgO SHNOs with negligible PMA (*19*).

**Fig. 4:**
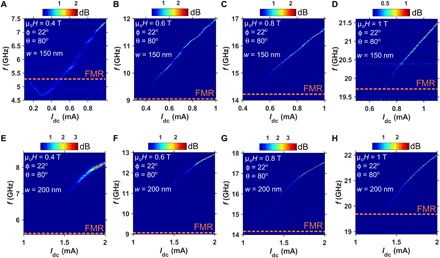
Current tunability of SW auto-oscillations at different OOP applied field strengths. PSDs of the SW auto-oscillations versus current for a *w* = 150 nm SHNO (**A** to **D**) and a *w* = 200 nm SHNO (**E** to **H**) subject to four different OOP field strengths (0.4, 0.6, 0.8, and 1.0 T). Orange dashed lines indicate the FMR frequency.

Having demonstrated the ability of nanoconstriction SHNO devices to generate propagating SWs, we now present the current tunability of the propagating mode under fixed OOP field strengths. Figure 4 (A to D) shows the current-dependent PSD plots for a 150-nm-wide SHNO. At 0.4 T, we observe a nonmonotonic current dependence accompanying a red shift in the auto-oscillation frequency at lower currents followed by a blue shift at higher currents. The FMR frequency, as shown by the dashed line, distinguishes the two opposite regimes of localized SWs versus propagating SWs. The nonmonotonic behavior at lower fields is a manifestation of a gradual change in confinement potential of the auto-oscillation mode with current and is consistent with theoretical predictions (*24*) and our simulation results discussed below. Qualitatively, with increasing current, the magnetization vector precesses with an increasingly large angle, which reduces the demagnetizing field via the projection of the magnetization along the static direction. This, in turn, leads to an effective reduction of the magnetization *M*_S_ in Eq. 1 and therefore changes the nonlinearity from a negative value to a positive one (*41*).

At higher fields, 0.6 T < μ_0_*H* < 1 T, only a blue-shifted behavior is observed, highlighting the dominance of large positive N caused by the PMA. Note that the variation of auto-oscillation frequency with electric current results in a very large positive value of the current tunability (*df*/*dI*), reaching values over 4 GHz/mA in our devices (see Fig. 4B). The current dependence for the wider (200 nm) SHNO, shown in Fig. 4 (E to H), only exhibits a blue-shifted auto-oscillation frequency starting from the threshold current in all fields, 0.4 T ≤ μ_0_*H* ≤ 1 T. Note that we no longer observe any auto-oscillation localization in the wider nanoconstriction, even at the lowest field of 0.4 T, which is consistent with stronger PMA in wider nanoconstrictions. We also emphasize that the spectral linewidth of auto-oscillations Δ*f* < 20 MHz, extracted using a Lorentzian fit, yields a quality factor *Q* = *f*/Δ*f* of up to 1000, indicating a considerably higher degree of oscillation coherence of the generated propagating SWs in our devices. In addition, our demonstration does not require very large values of PMA field to excite propagating SWs, while the generation takes place in a wider frequency spectrum ranging from 3 to 22 GHz (*20*).

### Micromagnetic simulations

**Fig. 5:**
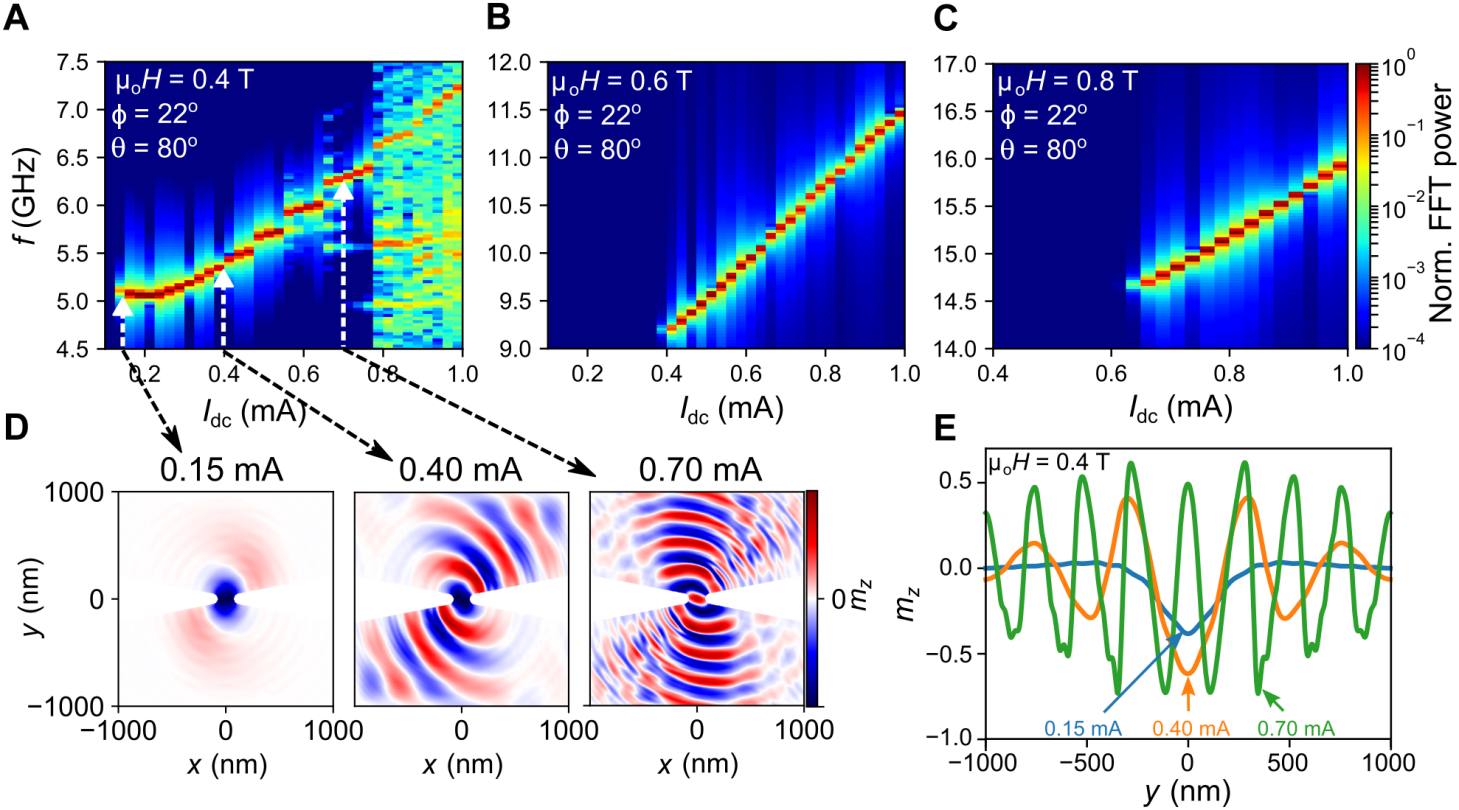
Micromagnetic simulations. (**A** to **C**) Micromagnetically simulated PSDs as a function of applied dc through a 150-nm nanoconstriction under field conditions used in the experiments. FFT, fast Fourier transform. (**D**) Snapshots of the instantaneous *m_z_* component at three different currents showing how the auto-oscillations transition from localized (0.15 mA) to propagating (0.4 and 0.7 mA) with a wave vector that increases with current. (**E**) Cuts through (D) along the *y* axis at the three different currents.

Last, we present micromagnetic simulations performed using comparable conditions as in our electrical measurements to study the spatial profiles of the SW auto-oscillations for a 150-nm nanoconstriction SHNO. All the magnetodynamical parameters used in the simulations are directly taken from the ST-FMR measurements discussed above. Figure 5 (A to C) shows the current-dependent PSD under three different fixed OOP field strengths, indicating excellent agreement with our experimental results in Fig. 4 (A to C). At 0.4 T, we observe a similar nonmonotonic current dependence of the auto-oscillation frequency with simulated current, starting with a red-shifting frequency followed by a blue-shifted behavior (see Fig. 5A). It is interesting to note the appearance of multiple frequency steps at the lowest field. These are likely related to discreet changes in the wave vector and can also be observed in our experimental results at the same field (Fig. 4A), albeit as smoother transitions. At higher applied fields, these mode transitions disappear both in the experiments and in the simulations. The auto-oscillations then only show a blue-shifted frequency behavior.

To gain deeper insight into the nonmonotonic frequency behavior, we plot the spatial profiles of the simulated auto-oscillations at three representative currents in Fig. 5D. The nature of the auto-oscillations is qualitatively different at low and high current: In the low-current region, where *df*/*dI* < 0, the auto-oscillations are clearly localized to the vicinity of the nanoconstriction; at the two higher currents, where *df*/*dI* > 0, the SWs clearly propagate with a wave vector that increases with current. Figure 5E shows snapshots of the instantaneous *m_z_* component at the three different currents, highlighting the transition from a localized nature at 0.15 mA to propagating SWs with about twice as large wave vector at 0.70 mA compared to 0.40 mA.

We also analytically calculated the propagation lengths for the SWs shown in Fig. 3 (A and B) at μ_0_*H* = 0.5 T as ξ=vg/(αω), where ω is the angular frequency of the SWs and vg=∂ω/∂k is the SW group velocity. Using the dispersion relation of Eq. 3 (see Materials and Methods), we estimate *k* = 2.2 × 10^7^ *m*^−1^ and *v*_g_ = 1297 m/s for the 150-nm SHNO device and *k* = 2.66 × 10^7^ m^−1^ and *v*_g_ = 1552 m/s for the 200-nm one, which gives us correspondingly ξ = 1.1 and ξ = 1.2 μm, which is in good agreement with our micromagnetic simulation results shown in Fig. 5E. Note that despite higher damping in our SHNOs, these propagation lengths are of the same order of magnitude reported in (*20*), owing to larger group velocities estimated in our case. For practical magnonic applications and also for improved mutual synchronization of neighboring nanoconstrictions, these propagation lengths should likely be sufficient.

To further extend the SW propagation, a number of different approaches could be investigated. As a large part of the SW damping originates from spin pumping into the W layer, one could fabricate the SHNO in such a way that the CoFeB/W interface only exists in the active nanoconstriction region. Alternatively, one could narrow the width of the region outside of the nanoconstriction to benefit from the spin current from the W layer, as well as outside of the auto-oscillating region [see, e.g., Fig. 5C in (*16*)], which can markedly extend the SW propagation (*6*, *7*, *42*).

## DISCUSSION

The capability to generate high-frequency SOT-driven coherent propagating SWs in metal-based CMOS-compatible SHNO devices has particular potential for a number of reasons. First, the SW generation takes place already at very small operational currents with thresholds as low as 0.15 mA, with a straightforward development path toward even lower currents, making these oscillators the most amenable to adaptation in nanomagnonic circuits. Thanks to the large SHA provided by the β-W layer together with the strong PMA due to thinner CoFeB layer, the critical threshold current density required to excite propagating SWs with SOT in metal devices has been reduced by about two orders of magnitude (10^7^ A/cm^2^) compared to theoretical predictions based on Pt and zero PMA (*40*). Further reduction of the critical current density should also be possible by yet higher PMA in yet thinner CoFeB. Second, their current tunability should allow these SHNO to rapidly switch between localized and propagating SWs, effectively acting as tunable nanoscale sources of ultrashort SW pulses and wave packets (*26*). Third, the wave nature of the propagating SWs will now allow for experimental realizations of SOT-driven SW computing (*25*) such as interference-based majority gates. Last, the propagating SW modes could further boost the long-range mutual synchronization of SHNO chains and networks for neuromorphic computing applications. Given the recent experimental demonstration of neuromorphic vowel recognition using four mutually synchronized spin-torque nano-oscillators (*22*), we believe that the long-range mutual synchronization driven by propagating SWs in low–operational current SHNOs may turn out to be the most viable solution for scaling neuromorphic computing to large dynamical neural networks.

## MATERIALS AND METHODS

### Nanoconstriction device fabrication

A trilayer stack of W(5)/Co_20_Fe_60_B_20_(1.4)/MgO(2) (thicknesses in nm) was grown at room temperature on an intrinsic high-resistivity Si substrate (ρ_Si_>10 kilohm·cm) using an AJA Orion 8 magnetron sputtering system. dc and RF sputtering were sequentially used for the depositions of metallic and insulting layers, respectively. The chamber was evacuated to a base pressure of 2 × 10^−8^ mtorr, while the Ar gas pressure was maintained at 3 mtorr during the growth of all the layers. The deposition rate of W was kept at 0.09 Å/s to obtain high-resistivity β-phase exhibiting a large SHA (*19*). The same deposition rate was maintained for the Co_20_Fe_60_B_20_ layer, while the MgO layer was grown at 0.04 Å/s. The as-deposited stack was subsequently annealed under chamber base pressure at 300°C for 1 hour to induce PMA. The annealing process was followed by deposition of 4 nm of SiO*_x_* to protect the MgO layer from degradation due to exposure to the ambient conditions. The resistivities of β-phase W and Co_20_Fe_60_B_20_ were measured as 213 and 100 microhm·cm, respectively. The trilayer stack was then patterned into an array of rectangular mesas with dimensions of 4 μm by 14 μm, and the nanoconstriction SHNO devices with different widths were defined at the center of these mesas by a combination of electron beam lithography and argon ion beam etching using negative electron beam resist as the etching mask. In addition, we patterned microstrips with dimensions of 6 μm by 18 μm for ST-FMR measurements. The fabrication process was detailed in (*19*).

### Analytical calculation of nonlinearity coefficient for a thin magnetic film

To calculate the nonlinear coefficient N, shown on Fig. 1B, we start from the magnetic energy density, which consists of Zeeman, dipolar, and PMA terms. Using a well-known method (*27*), in which the Hamiltonian is expressed in the elliptically polarized dimensionless variables by sequentially applying Holstein-Primakoff and Bogoliubov transformations with the further elimination of the nonresonant three-waves processes, one can derive the final result [see (*43*) for the details] as

$$N=\frac{2\omega_{0}}{A}(T-3\frac{{|W_{1}|}^{2}+{|W_{2}|}^{2}}{\omega_{0}})$$ (1)

where

$$\begin{array}{l} T=[3{\left(u^{2}+{|v|}^{2}\right)}^{2}-1]U_{1}/2-3u(u^{2}+{|v|}^{2})(vU_{2}+v^{*}U_{2}^{*})\\ W_{1}=3(u^{2}+{|v|}^{2})(uV-v^{*}V^{*})/2-(uV+v^{*}V^{*})/2\\ W_{2}=-{\mathrm{uv}}^{*}(uV-v^{*}V^{*}) \end{array}$$

The FMR frequency, shown by a solid line on Fig. 3, can be calculated as

$$\omega_{0}=\sqrt{A^{2}-{|B|}^{2}}$$ (2)

The coefficients of Bogoliubov transformation are

$$u=\text{sign}(A)\sqrt{\frac{A+\omega_{0}}{2\omega_{0}}},\;v=\frac{B^{*}}{|B|}\sqrt{\frac{A-\omega_{0}}{2\omega_{0}}}$$

where

$$\begin{array}{l} A=\omega_{H}-\frac{1}{2}(\omega_{k}-\omega_{M}){\text{cos}}^{2}\theta_{M}\\ B=-\frac{1}{2}(\omega_{k}-\omega_{M}){\text{cos}}^{2}\theta_{M}\\ V=(\omega_{M}-\omega_{k})\text{sin}\theta_{M}\text{cos}\theta_{M}\\ U_{1}=(\omega_{k}-\omega_{M})\text{sin}\theta_{M}(\frac{3}{2}{\text{cos}}^{2}\theta_{M}-1)\\ U_{2}=-B/2 \end{array}$$

In the above expressions, we used the notations ω_M_ = γμ_0_*M*_S_, $\omega_{k}={\gamma \mu }_{0}H_{k}^{⊥}$, and ω_H_ = γμ_0_*H*_M_, where *M*_S_ is the saturation magnetization, $H_{k}^{⊥}$ is the PMA field, and *H*_M_ is the effective internal field. The latter can be defined together with the internal angle of magnetization θ_M_ from the following equations

$$\begin{array}{l} H_{M}\text{cos}\theta_{M}=H_{\text{ex}}\text{cos}\theta_{\text{ex}}\\ (H_{M}-H_{k}^{⊥}+M_{S})\text{sin}\theta_{M}=H_{\text{ex}}\text{sin}\theta_{\text{ex}} \end{array}$$

where *H*_ex_ is the externally applied magnetic field applied at the OOP angle θ_ex_.

The spectrum of the propagating SWs in the linear limit is defined as (*34*)

$$\omega (k)={\gamma \mu }_{0}\sqrt{\left(H_{\text{int}}+{\mathrm{Dk}}^{2})(H_{\text{int}}+M_{\text{eff}}\text{cos}\theta_{\text{int}}+{\mathrm{Dk}}^{2}\right)}$$ (3)

where *D* = 2*A*_ex_/(μ_0_*M*_eff_) is the dispersion coefficient and *A*_ex_ is the exchange stiffness constant.

### ST-FMR measurements

The magnetodynamical parameters of the devices under investigation were determined by performing ST-FMR measurements at room temperature on a microstrip, with dimensions of 6 μm by 18 μm, of a W(5)/Co_20_Fe_60_B_20_(1.4)/MgO(2) stack. An RF current modulated at 98.76 Hz was injected through a high-frequency bias-T through the microstrip at a frequency characteristic of FMR (3 to 12 GHz), generating SOTs and Oersted field under an external applied IP magnetic field. The resulting torques excite the magnetic moment to precess in the CoFeB layer, leading to a time-dependent change in the resistance of the microstrip due to the MR of the CoFeB layer (*36*). The oscillating MR mixes with the RF current to create a dc voltage, *V*_mix_, across the microstrip and was measured using the circuit displayed in the inset of Fig. 2A. All ST-FMR measurements shown in Fig. 2 were carried out by sweeping an IP field (φ = 30°) from 350 to 0 mT, while the frequency of the input RF signal was kept fixed. To determine the SHA, we injected small dc currents in addition to the RF current through the dc and RF ports of a bias-T, respectively. The resonance feature in voltage response from each field sweep was fitted to a sum of one symmetric and one antisymmetric Lorentzian sharing the same resonance field and linewidth. In Fig. 3, ST-FMR measurements on the microstrip were performed by sweeping the applied field at a fixed IP angle of φ = 22° and OOP angle of θ = 80° to measure the FMR frequency under the identical conditions used during auto-oscillation measurements.

### Microwave measurements

All microwave electrical measurements were carried out at room temperature using a custom-built probe station with the sample mounted at a fixed IP angle on an OOP rotatable sample holder between the pole pieces of an electromagnet producing a uniform magnetic field. A direct positive electric current, *I*_dc_, was provided through the dc port of a high-frequency bias-T, and the resulting auto-oscillating signal was then amplified by a low-noise amplifier with a gain of ≥32 dB and subsequently recorded using a spectrum analyzer from Rohde & Schwarz (10 Hz to 40 GHz) comprising a low-resolution bandwidth of 300 kHz. We measured multiple SHNO devices and restricted the maximum current to 1 mA for 150 nm and 2 mA for 200-nm nanoconstriction devices to avoid irreversible changes due to device degradation in the output microwave characteristics.

### Micromagnetic simulations

The micromagnetic simulations were performed using the graphics processor unit–accelerated program mumax^3^ (*44*) with input provided by the COMSOL simulations. A 150-nm-wide nanoconstriction SHNO device was modeled into 1024 by 1024 by 1 cells with an individual cell size of 3.9 nm by 3.9 nm by 1.4 nm. The material parameters used in simulations such as the saturation magnetization μ_0_*M*_S_= 0.93 T, the gyromagnetic ratio γ/2π = 29.9 GHz/T, and the Gilbert damping constant α = 0.023 were obtained from ST-FMR measurements on a microstrip. We used a PMA field of $\mu_{0}H_{k}^{⊥}$= 0.57 T, slightly lower than the measured value of 0.62 T on a microstrip. The exchange stiffness constant of *A*_ex_ = 19 × 10^−12^ J/m for CoFeB was taken from (*45*). The distribution of charge current density and the resulting Oersted field landscape for a W/CoFeB bilayer was obtained with COMSOL simulations. The corresponding spin current was then estimated from the simulated charge current in the W layer, generating a transverse pure spin current along the OOP direction while considering an SHA of θ_SH_= −0.41, obtained from ST-FMR measurements on a microstrip. The magnetization dynamics was simulated by integrating the Landau-Lifshitz-Gilbert-Slonczewski equation over 62.5 ns, with the first 31 ns discarded in the following analysis to exclude transient effects. The auto-oscillation spectra were obtained by performing the fast Fourier transform of the simulated time evolution of the magnetization averaged over sample volume. The full spatial maps of the magnetization were extracted from the time domain data at a fixed time of 32 ns.

## Supplementary Material

http://advances.sciencemag.org/cgi/content/full/5/9/eaax8467/DC1

Download PDF

Spin-orbit torque–driven propagating spin waves

## SUPPLEMENTARY MATERIALS

Supplementary material for this article is available at http://advances.sciencemag.org/cgi/content/full/5/9/eaax8467/DC1 Determination of SHA using linewidth analysis
