# Supplementary material for: Spin-orbit torque–driven propagating spin waves
Source: Sci Adv. 2019 Sep 27;5(9):eaax8467. doi: 10.1126/sciadv.aax8467 (PMC6868678; doi:10.1126/sciadv.aax8467)
Supplement: http://advances.sciencemag.org/cgi/content/full/5/9/eaax8467/DC1 [file supp_5_9_eaax8467__index.html]

Science Advances | Science AdvancesAAASSearchScience AdvancesMenu

## Supplementary Materials

**This PDF file includes:**

- Determination of SHA using linewidth analysis

Download PDF

**Files in this Data Supplement:**

- Adobe PDF - aax8467\_SM.pdf
