# Supplementary material for: Spin-orbit torque–driven propagating spin waves
Source: Sci Adv. 2019 Sep 27;5(9):eaax8467. doi: 10.1126/sciadv.aax8467 (PMC6868678; doi:10.1126/sciadv.aax8467)
Supplement: Download PDF [file aax8467_SM.pdf]

Science Advances
AAAS

advances.sciencemag.org/cgi/content/full/5/9/eaax8467/DC1

Supplementary Materials for

Spin-orbit torque–driven propagating spin waves

H. Fulara, M. Zahedinejad, R. Khymyn, A. A. Awad, S. Muralidhar, M. Dvornik, J. Åkerman*

*Corresponding author. Email: johan.akerman@physics.gu.se

Published 27 September 2019, *Sci. Adv.* **5**, eaax8467 (2019)
DOI: 10.1126/sciadv.aax8467

This PDF file includes:

Determination of SHA using linewidth analysis

Determination of SHA using linewidth analysis

The spin Hall angle (SHA) based on the linewidth analysis is described by the following equation[36]

$$\theta_{SH} = \frac{\delta\Delta H/\delta I_{dc}}{\frac{2\pi f}{\gamma}\frac{\sin\varphi}{(H_{ext}+0.5M_{eff})\mu_0 M_s t}\frac{\hbar}{2e}}\frac{R_{CoFeB}+R_W}{R_{CoFeB}}A_C$$

where ΔH is the linewidth extracted as HWHM from the ST-FMR resonance peak, t is the thickness of the CoFeB layer, e is the electron charge, $\hbar$ is the Planck's constant, R_{CoFeB} and R_W are the resistances of the CoFeB and W layers, respectively, and A_C is the cross-sectional area of the measured microstrip.

To determine the spin Hall angle θ_{SH}, we then measured the current-induced linewidth changes for two opposite in-plane field orientations at a fixed microwave frequency of 7 GHz. The inset of Figure 2B shows the linewidth dependence on dc current, with positive current providing a negative damping when the field is along $\phi = 30°$ (blue squares), and positive damping when the field direction is reversed to 210° (red dots). We fit the linewidth variation to a linear equation for both field polarities and extracted the slope value of 1.48 mT/mA. By substituting the extracted slope value along with other material parameters in the above stated equation, we obtain a high value of SHA, $\theta_{SH} = -0.41$, comparable to previous studies and characteristic of highly resistive β-W [19,36].
